# Physical health effects of sedentary behaviour on adults with an intellectual disability: A scoping review

**DOI:** 10.1177/17446295221107281

**Published:** 2022-06-12

**Authors:** Louise Lynch, Mary McCarron, Jessica Eustace-Cook, Éilish Burke, Phillip McCallion

**Affiliations:** IDS‐TILDA, School of Nursing and Midwifery, 8809 Trinity College, Dublin, Ireland; IDS‐TILDA, School of Nursing and Midwifery, 8809 Trinity College, Dublin, Ireland; IDS‐TILDA, School of Nursing and Midwifery, 8809 Trinity College, Dublin, Ireland; IDS‐TILDA, School of Nursing and Midwifery, 8809 Trinity College, Dublin, Ireland; School of Social Work, College of Public Health, Temple University, Philadelphia, PA, USA

**Keywords:** sedentary behaviour, adults, intellectual disability

## Abstract

This literature review was designed to establish the effects of sedentary behaviour on the physical health of adults with an intellectual disability. Sedentary behaviour is defined as any waking behaviour characterized by an energy expenditure of ≤1.5 METs while in a sitting, lying or reclining posture. An extensive search was executed in six databases: EMBASE, Medline, CINAHL, PsycINFO, ASSIA and Web of Science. Following screening, 18 articles remained for inclusion in the review. A thematic analysis using the Braun and Clarke six step process resulted in the identification of seven broad health areas. Studies showed a prevalence of obesity, multimorbidity and metabolic syndrome as well as elevated levels of sedentary behaviour in adults with an intellectual disability. This literature review demonstrated that sedentary behaviour could be a contributor to the poor health which is common in adults with an intellectual disability. However to date the body of evidence does not confirm a cause-and-effect relationship.

## Introduction

In order to understand the physical health effects of sedentary behaviour, it is important to understand what health is and what it means to be healthy. The constitution of the World Health Organisation (WHO) states that health does not just refer to the absence of disease but is a complete state of social, physical and mental well-being and represents a fundamental right for all (World Health Organisation, 1995). Conversely, a cross-sectional study found that people with physical disabilities felt their disability did not define their state of health but rather that an absence of illness was what made them healthy (Nazli, 2012). The perception of being healthy does not necessarily correspond with reports on health conditions. Over 73% of adults with an intellectual disability who participated in The Intellectual Disability Supplement to the Irish Longitudinal Study on Aging (IDS-TILDA) perceived their health as “very good” or “good” despite multimorbidity levels in excess of 71% (Lynch, 2021; McCarron et al., 2013). This perception of good health was retained and sometimes exceeded across the 10 years of the study (Lynch, 2021; McCarron, 2011; McCarron et al., 2014, 2017). In general adults with an intellectual disability have shorter life spans than their counterparts in the general population, dying approximately 19 years earlier and from different causes (Cooper et al., 2020; McCarron et al., 2015). A Brazilian proxy-based study of adults aged 35–60 years showed that after 5 years, despite the relatively young age profile, adults with intellectual disability had more physical and mental health issues than controls (Guilhota et al., 2016). Worldwide, Ischaemic heart disease was the primary cause of death in the general population (Khan et al., 2020). A leading contributor to heart disease is overweight and obesity which was observed in almost 80% of a cohort of adults with intellectual disability but although these Cardiovascular disease (CVD) risk factors were prevalent, heart disease was not the main cause of death (McCarron et al., 2017). Instead, for adults with an intellectual disability choking and respiratory infections were the leading causes of death (Cooper et al., 2020; World Health Organisation, 2020). However the shorter lifespan combined with the considerable multimorbidity rates observed in the intellectual disability population compared to the general population warrant further research and attention to cardiovascular risk factors such as sedentary behaviour (SB) and physical inactivity are essential until a greater understanding of these behaviours is obtained.

While both SB and physical inactivity are typified by low energy expenditure they are different and should be addressed separately. SB has been defined as ‘any waking behaviour characterized by an energy expenditure of ≤1.5 METs while in a sitting, lying or reclining posture’ for example watching television or working on a computer while physical inactivity is not achieving the WHO recommended activity levels for health (Tremblay et al., 2017; World Health Organisation, 2016). A review of systematic reviews on the effects of SB on health found that SB could be an important contributor to health irrespective of physical activity (PA) levels (Rezende et al., 2014). In fact in the general population, there is evidence to suggest that increased sedentary time is associated with greater risks for all-cause mortality, an increase in metabolic risk factors, the incidence of CVD, type 2 diabetes risks and certain types of cancer (Biswas et al., 2015; Chomistek et al., 2013; Edwardson et al., 2012; Krishnan et al., 2009; Lynch et al., 2018). Hence while studies on the general population indicate that there may be health issues associated with increased time in SB, only limited information is available for adults with an intellectual disability. The aim of this literature review is to understand the physical health effects of sedentary behaviour in the adult intellectual disability population.

## Methods

### Research question

The PICO approach was used to define the research question for this literature review as follows:

• P [Population or problem]: Adults aged 18+ with an Intellectual Disability
• I [Intervention or exposure]: Sedentary behaviour (SB) level in line with the definition of SB
• C [Comparison]: Individuals with all levels of intellectual disability living in residential, institutional or hospital settings, community group homes, with family or independently
• O [Outcome]: Physical health effects of SB

The research question to be addressed was:

“What are the physical health effects of sedentary behaviour on adults with an intellectual disability?”

### Search

The article eligibility criteria used for this review is summarised in Table 1. This eligibility criteria was formulated using PICO. Although the focus of the review is SB, both SB and inactivity were included in the search concepts to ensure no articles were omitted.

**Table 1.** Article eligibility criteria.

	**Inclusion criteria**	**Exclusion criteria**
Population	Adults aged 18+ with an intellectual disability	All children and adults without an intellectual disability
Study design	RCTs, cohort, case-control and qualitative	Reviews of any type
Language	English	Non-English
Subject	Health effects of SB or inactivity	No reference to SB or inactivity and health
Timeframe	None up to April 2021	None

#### Information sources

A subject librarian assisted with performing the search for this literature review. The following six databases were searched:

• EMBASE
• Medline
• CINAHL
• PsycINFO
• ASSIA
• Web of Science

In addition, any reviews about health and sedentary behaviour that were discovered during the search process were searched for appropriate references.

#### Search strategy

The search strategy was refined using PICO and executed using three concepts. Concept 1 (C1) was sedentary behaviour and physical inactivity. Concept 2 (C2) was intellectual disability and learning disability. Concept 3 (C3) was impact, result, consequence, effect or response. All six databases were searched for each concept using MESH terms and keywords and the Boolean operator OR was applied to broaden the search. An example of the search string used for Embase is shown in Table 2. The results of each concept search were then combined using the Boolean operator AND. This was repeated in the other databases. This combined article list was then screened for final article inclusion.

**Table 2.** Embase search string.

**Concept**	**Index**	**Keywords**
C1: sedentary behaviour and physical inactivity	“Sedentary lifestyle”/exp	“sedentary life*” OR “sedentary behavi*” OR “physical* inactiv*” OR “inactive life*“ OR “immobile life*” OR sitting OR “reclin* posture*” OR “lying posture” OR “sedentary time*”
C2: intellectual disability and learning disability	“Intellectual impairment”/exp OR “mental deficiency”/exp OR “mentally disabled person”/exp OR “intellectual impairment”/exp OR “mental deficiency”/exp	“intellectual disabilit*” OR “intellectually disab*” OR “learning disabilit*” OR “mental handicap*” OR “mental retard*” OR “mentally disabl*' OR “learning disorder*” OR “mental deficien*” OR “mental deficit*” OR “mental* impair*” OR “intellectual impair*” OR “Trisomy 21*” OR “down syndrome*” OR “down syndrome*” OR “down/ s syndrome*” OR “downs syndrome*” OR “Trisomy 21*” OR Mongol* OR “down disease*” OR “downs syndrome” OR “translocation 15 21 22” OR “trisomy 21” OR “trisomy 21” OR “intellectual impair*” OR “intellectually impar*” OR “intellectual deficit*” OR “intellectual dysfunction” OR “Coffin-Siris Syndrome” OR “Cri-Du-Chat Syndrome” OR “de Barsy Syndrome” OR “de Lange Syndrome” OR “down Syndrome” OR “Kleefstra Syndrome” OR “Prader-Willi Syndrome” OR “Schinzel-Giedion Syndrome” OR Phenylketonuria
C3: impact, result, consequence or response	No appropriate index terms	Impact* OR result* OR outcome* OR upshot* OR respons* OR conseq* OR conclus* OR effect* OR affect* OR aftermath* OR repercuss* OR eventual OR aftereffect* OR finish* OR fallout OR react* OR “follow up*” OR follow-up* OR chall* OR detriment* OR barrier*

#### Screening process

**Figure 1.**
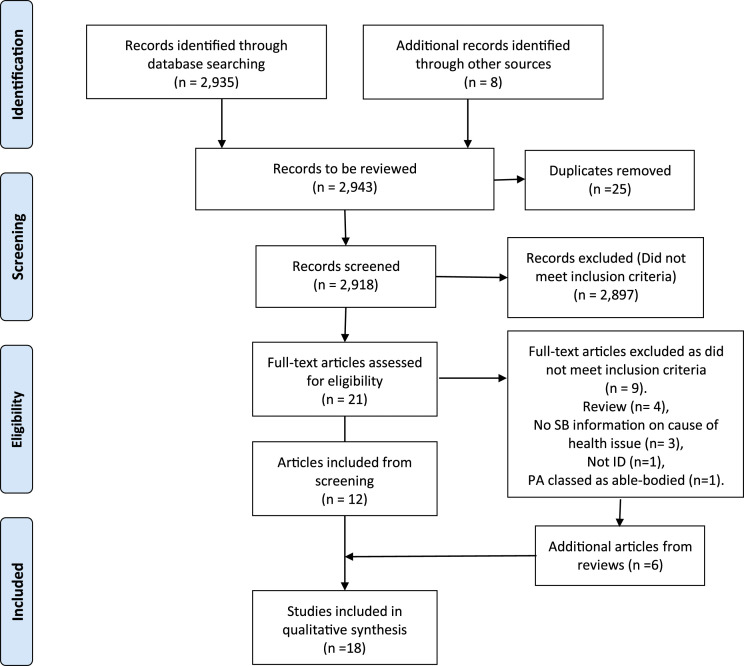
Prisma search.

A PRISMA flow diagram was used to illustrate the article screening process. See Figure 1. The articles output from the search were imported into Covidence, which was used as the screening management tool. Duplicates were removed and all articles were screened by title and abstract by the assessor, who then executed a full article review on 27 articles. These 27 articles consisted of 6 articles unearthed from reviews and 21 articles from the original search. This screening process was verified by another assessor. Data from the 27 articles was summarised in an excel spreadsheet. The headings used in this data extraction spreadsheet are shown in Table 3.

**Table 3.** Data extraction spreadsheet headings.

Article No
Article detail
No of participants
Study focus
Assessment type
Measurement device
Country
Age
Gender
Level of intellectual disability
Year
Study type
Outcome
Statistical results
Living arrangements
Sedentary or inactivity
Conclusions
Findings
Comment

#### Data synthesis

A qualitative synthesis of the final articles was performed and a thematic analysis using the Braun and Clarke Six Step process was completed as shown in Table 4 (Braun and Clarke, 2006). The first step involved the researcher building familiarity with the articles and data through several readings of each article, identifying the main aspects of each study and creating and completing the data extraction tool. This enabled a succinct review for each article in order to conduct step two. As patterns emerged and the topics became evident the researcher used colour coding to highlight similar topics and then moved to identify the broader themes. These themes were fine-tuned until seven umbrella themes remained.

**Table 4.** Six step thematic analysis process.

**Step number**	**Process**	**Explanation**
1	Data familiarisation	Complete data immersion
2	Generate initial codes	Topics, patterns of data
3	Search for themes	Broader theme identification
4	Review of themes	Theme refinement
5	Define and name themes	Categorise. Include sub-themes if required
6	Produce report	Complete write-up

## Results

A total of 18 articles were successfully screened into this review. These articles are summarised in Table 5. Overall, seven broad health themes were identified using the Braun and Clarke process as shown in Table 6, which shows the data source for the study, the number of participants and whether physical activity (PA)/inactivity or sedentary behaviour (SB) were used as determinants. Some overlap in themes may be observed, for example obesity is a risk factor in metabolic syndrome and multimorbidity. Although inactivity is different to SB, it was included in the search to ensure that all relevant studies were captured because the terminology has been inconsistently used in articles before the definition for SB was refined (Tremblay et al., 2017). Sedentary behaviour was used in eight articles to compare to physical health outcomes, with only three articles solely using SB for comparison, while physical inactivity or PA levels were considered in 15 articles. Thus the exclusion of inactivity or PA in this review would have resulted in an incomplete description of the state of the science, as over 50% of the articles would have been excluded. Eleven articles utilised some form of physical or objective measurement. The number of participants with intellectual disability represented in the articles in this review was 9,830.

**Table 5.** Final article summary.

**Article No**	**Article detail**	**No of Participants**	**Study Focus**	**Assessment type/measurement**	**Strengths and/or weaknesses**	**Country**
1	Wells, M. B., Turner, S., Martin, D. M. and Roy, A., 1997. Health gain through screening — coronary heart disease and stroke: developing primary health care services for people with intellectual disability. Journal of Intellectual and developmental Disability, 22(4), pp. 251–263.	120	Review of risk factors related to cardiovascular disease among people with intellectual disabilities	Health check and questionnaire with focus on family history and other CVD risk factors. Questionnaire also used to assess PA levels	No objective activity measurement, not a validated PA questionnaire and no information on amounts of time spent being active or inactive	UK
2	Havercamp, S.M., Scandlin, D. and Roth, M., 2004. Health disparities among adults with developmental disabilities, adults with other disabilities, and adults not reporting disability in North Carolina. Public health reports, 119(4), pp. 418–426.	946 in intellectual disability group. (1598 in disability group and 4358 in no disability group). 6902 total.	Purposes of this study were to identify disparities between adults with developmental disabilities and non-disabled adults in health and medical care and compare this pattern of disparities to the pattern of disparities between adults with other disabilities and adults without disabilities.	Data on the health of adults with developmental disabilities living in North Carolina (NC) were compared with data from the 2001 NC Behavioral Risk factor Surveillance System (BRFSS) survey, a random telephone survey of adults and the NC national core indicator survey	Large sample size which covered all levels of intellectual disability but due to telephone survey may not be fully representative. No objective measurements and no quantifable amounts of PA.	USA
3	Carmeli, E., Bachar, A. and Barchad, S., 2007. Biochemical assessments of total antioxidant status in active and nonactive female adults with intellectual disability. Research in Sports Medicine, 15(2), pp. 93–101.	21	To compare relationship between long term PA and inactivity and plasma antioxidative status i.e. vitamin and enzyme levels in female adults with an intellectual disability	The antioxidant defence system measured was Superoxide Dismutase (SOD), Catalose (CA), Gluthathiona Peroxidase (GPX), vit a and vitamin E. Chromosystem with UV detector, liquid chromatography and tests by Randox labs	Small sample size and focussed on females. Compared sedentary controls with PA group. All from residential care setting. No details on level of activity or ID specified. All measured results were significantly better in active group (*P* < 0.05)	Israel
4	de Winter, C. F., Magilsen, K. W., van alfen, J. C., Penning, C., and Evenhuis, H. M. (2009). Prevalence of cardiovascular risk factors in older people with intellectual disability. American Journal on Intellectual and Development disabilities, 114(6), 427–436.	470	Aims of (a) determining the prevalence of cardiovascular risk factors in older people with intellectual disability and (b) identifying the relation of these occurrences with down syndrome and level of intellectual disability	Medical history through interview and chart review. Also physical examination	Large sampe size but skewed towards females (73%/27%). 68.3% did no exercise i.e. did not do 150mins of MVPA per week. All intellectual disability levels but more severe and moderate. Did not talk about SB.	Holland
5	Moss, S. J., 2009. Changes in coronary heart disease risk profile of adults with intellectual disabilities following a physical activity intervention. Journal of Intellectual disability Research, 53(8), pp. 735–744.	100	To determine the CHD risk profile of adults with intellectual disability residing in a care facility and determine the effect of a PA intervention on the CHD risk profile of the residents.	Questionnaire and physical assessments of body fat, BMI, BP, non-fasting glucose and cholesterol, cardiorespiratory fitness test.	Active classed as MVPA min once per week. Most common risk factors for CVD were inactivity and overweight. Being active was 1 or more sessions of MVPA per week	South Africa
6	de Winter, C. F., Magilsen, K. W., van Alfen, J. C., Willemsen, S. P., and Evenhuis, H. M. 2010. Metabolic syndrome in 25% of older people with intellectual disability. Family Practice, 28 (2), 141–144.	470	Studied the prevalence of the metabolic syndrome in the older population with intellectual disability and its association with patient characteristics	Waist measurement, fasting blood sample, BP. Semi-structured interview to assess daily PA level. PA criteria was to meet WHO requirement 5 × 30 mins MVPA per week.	Large sample size which had significantly higher metabolic syndrome than general population. Mild intellectual disability even higher. No PA relationship detected. 3x more females than males due to setting being ex-nunnery. No SB measures	Holland
7	Haveman, M., Perry, J., Salvador-Carulla, L., Walsh, P.N., Kerr, M., van Schrojenstein Lantman-de Valk, H., Van Hove, G., Berger, D. M., Azema, B., Buono, S. and Cara, A. C., 2011. Ageing and health status in adults with intellectual disabilities: results of the European POMONA II study. Journal of Intellectual and developmental Disability, 36(1), pp. 49–60.	1253	1. What are the age-specific differences regarding type of residence, social relations, and state of employment for adults with intellectual disability in this European study? 2. Are there systematically and statistically significant differences between age groups with regard to lifestyle risk factors such as smoking, alcohol, and physical activity? 3. Which of the 17 medical conditions measured by the POMONA Checklist of Health Indicators (P15) are more prevalent in older age (55–64, 65 and older) compared to younger age groups?	The POMONA Health Interview Survey and Evaluation form (P15) comprises items related to demographic characteristics of respondents, health status, health determinants, and health systems.	51.8% of total sample sedentary but 65+ years 60.9% sedentary. 41.4% of total sample do light activites for 4 h per week. Good sample size but across european and cross-sectional not longitudinal. conclusions not generalisable as country samples were from pilots and unrepresentative of the intellectual disability population in each country	Cross-European study. Data from 14 countries

8	De Winter, C. F., Bastiaanse, L. P., Hilgenkamp, T. I. M., Evenhuis, H. M. and Echteld, M. A., 2012. Overweight and obesity in older people with intellectual disability. Research in developmental disabilities, 33(2), pp. 398–405.	945	Provide info on overweight and obesity on a large sample of people with intellectual disability looking at prevalence and assoication with various factors like physical activity	Physical activity assessed wearing pedometers for 2 weeks. >7500 steps classes as sufficient physical activity. Weight , height and waist-hip measurements and body fat taken	Part of large HA-ID study. Representative of population of older adults, 50+ years. Obesity 25.6% vs 9.6% in general population. Atypical antipsychotics associated with obesity	Holland
9	Vis, J. C., de Bruin-Bon, R. H., Bouma, B. J., Backx, A. P., Huisman, S. A., Imschoot, L. and Mulder, B. J., 2012. “The sedentary heart”: Physical inactivity is associated with cardiac atrophy in adults with an intellectual disability. International journal of cardiology, 158(3), pp. 387–393.	182	Aim of this study was to investigate Left ventricular volumes and mass in a physically inactive population of adults with an intellectual disability, including subjects with DS.	Echocardiography was performed with a portable GE VIVID I in all included subjects and physical activity was measured by a questionnaire, Short Questionnaire to Assess Health-enhancing physical activity (SQUASH)	Adults with and without DS compared to inactive controls. 115 DS vs 26 without DS.PA level in adults with intellectual disability was 2X less than controls.SQUASH form not validated for persons with intellectual disability. iLVM positively related to SQUASH score	Netherlands
10	Haider, S. I., Ansari, Z., Vaughan, L., Matters, H., and Emerson, E. 2013. Health and wellbeing of Victorian adults with intellectual disability compared to the general Victorian population. Research in developmental disabilities, 34(11), 4034–4042.	897	Aims are to describe and present results from the Victorian population Health Survey of people with an Intellectual disability 2009 (VPHS-ID 2009), the first population level survey in Victoria, Australia and to compare the health and wellbeing of people with Intellectual disability to the general Victorian population.	telephone interviews with proxy respondants	Administrative database used to identify participants may under-represent population. Sufficient PA was 150+ mins of MVPA. SB was 5X more in intellectual disability compared to general population.	Australia
11	Hsieh, K., Rimmer, J. H. and Heller, T., 2014. Obesity and associated factors in adults with intellectual disability. Journal of Intellectual disability Research, 58(9), pp. 851–863.	1450	Examined the relationships between nonmodifiable and modifiable risk factors/antecedents for obesity by addressing four primary research questions	Obesity determined by BMI. Hours of TV-watching per day. PA was measured with a question 'How many days a week is 30' MVPA done?'	Baseline data from Longitudinal Health and Intellectual disabilities study (LHIDS) but only 1 point in time. Obesity related to MVPA and no of hours watching TV. Morbid obesity 2X in younger adults.	USA
12	Geijer, J. R., Stanish, H. I., Draheim, C. C. and Dengel, D. R., 2014. Bone mineral density in adults with down syndrome, intellectual disability, and nondisabled adults. American Journal on intellectual and developmental disabilities, 119(2), pp. 107–114.	99 (but 33 non- intellectual disability)	Purpose of this study was to assess the BMD in adults with intellectual disability, with and without DS, and investigate whether BMD is associated with PA levels. Further aimed to determine whether differences exist in the BMD among three groups	BMD and activity levels. DXA scan and NHANES III physical Activity Survey was used to assess walking activity	Good cross-sectional, comparative design. Time walking was measure of PA used, bouts, minutes in bouts, total walking time and total PA. Total PA time significantly less in both intellectual disability groups. In DS group total bouts walking significantly related to BMD but not in others.	USA
13	Carmeli, E., Bachar, A., Rom, O. and Aizenbud, D., 2015. Oxidative stress and nitric oxide in sedentary older adults with intellectual and developmental disabilities. In Pathophysiology of Respiration (pp. 21–27). Springer, Cham.	23. 12 in sedentary group and 10 in active group	Aim of the study was to investigate the serum levels of global OS and NO metabolites (NOx) in sedentary and non-sedentary older adults with intellectual disability	6 mL of venous blood samples were collected after an overnight fasting and were drawn into “vacutainer” 10 mL tubes for metabolite assays	Focus on moderate to profound and living in residential care. Sedentary group bedbound or wheelchair so most of day spent in SB. Age range 50–60 years. Specificity of serum OS values questioned.	Israel
14	Bryant, L. D., Russell, A. M., Walwyn, R. E. A., Farrin, A. J., Wright‐Hughes, A., Graham, E. H., Nagi, D., Stansfield, A., Birtwistle, J., Meer, S. and Ajjan, R. A., 2018. Characterizing adults with type 2 diabetes mellitus and intellectual disability: outcomes of a case‐finding study. Diabetic Medicine, 35(3), pp. 352–359.	147	In this paper we present data characterizing the study population in terms of diabetes control, health, and access to diabetes management services and support.	Interview to establish diabetes management and supporter's role in diabetes management and identify preferences for assistance with diabetes management.	Participants had mild to moderate intellectual disability and living in the community. 14% did no activity and 26% did some every day. Intensity not assessed. People using insulin excluded.	UK
15	Harris, L., McGarty, A. M., Hilgenkamp, T., Mitchell, F. and Melville, C. A., 2018. Correlates of objectively measured sedentary time in adults with intellectual disabilities. Preventive medicine reports, 9, pp. 12–17.	152	Aim of this study is to add to the available evidence by investigating correlates of objectively measured sedentary behaviour in adults with intellectual disabilities.	A secondary analysis of pooled baseline data from two RCTs of lifestyle behaviour change programmes. One RCT focused on weight management (*n* = 50; Harris et al., 2015; Harris et al., 2017) and the second one on increasing PA	Objectively measured SB was 72.9% and obesity based on height/weight measures. Physical and mental problems significantly related to SB time. Health assessments by questionnaire.	Scotland, UK
16	Kim, J. Y., and Yi, E. S. 2018. Analysis of the relationship between physical activity and metabolic syndrome risk factors in adults with intellectual disabilities. Journal of Exercise Rehabilitation, 14 (4), 592–597	17	This study intends to measure the PA of adults with disabilities using an accelerometer to confirm the relationship of their activity levels with biochemical variables and examine variables that can predict physical activities to provide basic data for use in exercises and programs to improve the PA levels of disabled adults	PA measured using an actical. Muscular strength was measured using a digital dynamometer	Mild and moderate Intellectual disability levels. Avg age was 29.88 years. More men then women (13 vs 4). PA objectively measured. PA positively correlated with total cholesterol and LDL	Korea
17	Tyrer, F., Dunkley, A. J., Singh, J., Kristunas, C., Khunti, K., Bhaumik, S., Davies, M. J., Yates, T.E. and Gray, L. J., 2019. Multimorbidity and lifestyle factors among adults with intellectual disabilities: a cross‐sectional analysis of a UK cohort. Journal of Intellectual disability Research, 63(3), pp. 255–265.	920	The primary purpose of this study was to determine the prevalence of multimorbidity in a population of adults with intellectual disability. We also aimed to identify risk factors, including lifestyle factors, for multimorbidity in adults with intellectual disability.	anthropometric measures (height, weight and blood pressure), blood samples and additional demographic and lifestyle data. The case record form included collection of data on chronic conditions. Questionnaire for gathering SB and PA information	SB related to multimorbidity. PA assessed as < or > 5x per week. Sitting question answer of 'sometimes or never' meets guidance of not sitting for extended periods. Younger adults 57% multimorbid vs 4% in general population of similar age.	UK
18	Murthy, S. and Hsieh, K., 2021. Examining Association Between Reported High Cholesterol and Risk factors in Adults With Intellectual and developmental disabilities (IDD): A five-Year Follow-Up. Intellectual and developmental Disabilities, 59(2), pp. 112–122.	1618. 925 completed for all 5 years	This study examined whether being physically inactive, having obesity, and/or having diabetes were predictors of reported high cholesterol over a 5-year span among adults with intellectual and developmental disabilities. It also explored whether there were any mediating effects of diabetes on the relationship between obesity and high cholesterol	Longitudinal Health and Intellectual and Development Study (LHIDDS) survey. Proxy reporting.	Outcome measure of study was high cholesterol. Physical inactivity was a “1” or “0” where “0” was doing some form of MVPA. High cholesterol significantly increased with age.	US

**Table 6.** Health themes and measurement.

	Health condition	Article topic	Article author	Data source	No of parts	Activity or SB
1	Metabolic syndrome	Prevalence of metabolic syndrome	De Winter et al. (2010)	Anthropometric and interview	470	PA
		Metabolic syndrome risk factors	Kim and Yi (2018)	objective measurements	17	PA
2	Overweight and obesity	Overweight and obesity	De Winter et al. (2012)	Pedometer	945	inactivity
		Obesity and factors	Hsieh et al. (2014)	Survey	1,450	SB
3	Multimorbidity and diabetes	Multimorbidity	Tyrer et al. (2019)	Anthropometric, bloods and form	920	PA and SB
		Diabetes	Bryant et al. (2018)	interview	147	inactivity
4	Cardiovascular	CVD Risk factors	Wells et al. (1997)	Questionnaire and health check	120	PA
		CVD Risk factors	Moss (2009)	Measurements and questionnaire	100	PA
		CVD Risk factors	de Winter et al. (2009, 2010)	Interview and chart review	470	inactivity
		CVD Risk factor: Cholesterol	Murthy and Hsieh (2021)	Proxy reporting	1618	inactivity
		Cardiac atrophy	Vis et al. (2012)	Questionnaire and echocardiogram	182	SB
5	General health	Health status	Havercamp et al. (2004)	Telephone survey	946	PA
		Health status	Haveman et al. (2011)	Survey	1253	SB and inactivity
		Health status	Haider et al. (2013)	Proxy interview	897	SB and PA
		Correlates of sedentary time	Harris et al. (2018)	Objective measurements and question	152	SB
6	Bone mineral density	Bone mineral density	Geiger et al. (2014)	Survey and DXA scan	99	PA
7	Body oxidation	Antioxidant status	Carmeli et al. (2007)	Bloods	21	PA versus SB
		Oxidative stress and nitric oxide	Carmeli et al. (2015)	Bloods	23	SB versus PA

### Metabolic syndrome

Metabolic syndrome is the presence of a collection of coronary risk factors which increase the risk of heart disease, stroke and diabetes (Swarup et al., 2021). A Metabolic syndrome prevalence of 25% was found in a Dutch older intellectual disability population of 470 people but a significant relationship was not found between physical inactivity, as specified by the WHO, and metabolic syndrome (*p* = 0.26) (World Health Organisation, 2020). However, the observed levels of metabolic syndrome in this intellectual disability population were significantly higher than those observed in the general Dutch population, especially for those with mild intellectual disability but females were overrepresented by a ratio of 3:1 (De Winter et al., 2010). A Korean study with 17 participants with mild and moderate intellectual disability showed that metabolic syndrome risk factors of total cholesterol and LDL-cholesterol levels were significantly positively correlated to objectively measured physical activity (PA) levels. In addition, the study which had 3X more men than women, found that HbA_1c_ was a predictor for PA (Kim and Yi, 2018).

### Overweight and obesity

A Dutch study as part of the large cross-sectional study, Healthy Aging and Intellectual Disability (HA-ID), which investigated overweight and obesity in older adults with intellectual disability defined physical inactivity as taking <7,500 steps per day. Observed obesity levels were higher than in the general population (25.6% vs 9.6%) and a significant relationship between inactivity and obesity as measured by waist circumference and higher body fat levels, was found. These observed elevated levels of central obesity are a concern due to the potential increase of cardiovascular risks and link to Metabolic syndrome (De Winter et al., 2012). Engagement in moderate PA (MVPA) and hours spent watching TV were factors that were significantly associated with obesity levels in a longitudinal study on adults with intellectual disability. PA and sedentary levels were assessed by a single question and showed that almost one third of participants did not do any MVPA. While the obesity levels in the intellectual disability cohort ranged from 26 to 54% higher than those seen in the general population, the morbid obesity levels observed in the younger adults were twice that seen in the general population (Hsieh et al. 2014).

### Multimorbidity and diabetes

In a UK study multimorbidity, which was defined as having two or more chronic conditions which negatively impact health and had a prevalence of 61.2%, was found to have significant associations with self-reported SB and doing less activity in a week, in a group of adults with intellectual disability. Obesity was the most common multimorbid condition seen with over 68.5% of participants being either obese or overweight (Tyrer et al., 2019). Of concern is that younger adults with intellectual disability were more than 14 times likely to be multimorbid than similar age groups in the general population (57.1 vs 4%).

An interview-based cross-sectional study on 147 participants with mild to moderate intellectual disability and Type 2 diabetes who lived in the community, demonstrated that over 79% had additional co-morbidities: cardiovascular disease (40%) and high cholesterol (16%) were the most prevalent. SB levels were high with only 26% reporting some level of activity daily but intensities were not assessed. In addition, over 20% had higher than recommended HbA_1c_ levels and over 87% were overweight or obese (Bryant et al., 2018).

### Cardiovascular

Wells et al. (1997) used health check and PA questionnaires to garner PA levels and CVD risk factors in a cross-sectional study with 120 participants. Over 51% of intellectual disability participants had done some form of moderate intensity activity in the preceding 4 weeks, compared to over 93% in a control group while over 48% of the intellectual disability participants had done no moderate intensity activity. There was a greater incidence of high body mass index (BMI) in the intellectual disability group, who were significantly heavier than the control group. In addition several intellectual disability participants had abnormal cholesterol levels. The study concluded that people with intellectual disability had higher risk factors for CVD and stroke than the general population. However, the study had no objective activity measures, the PA questionnaire was not validated and there was no information available on the amount of time spent being active. Moss (2009) looked at changes in Coronary heart disease after a PA intervention to assess risk. Pre-intervention results indicated that over 85% of the participants were inactive (i.e. they did not do any MVPA) and 67% were overweight or obese. However being active was classified as doing one or more session of MVPA a week which does not comply with WHO guidelines. Higher than recommended levels of glucose concentrations were observed in 28% of participants which could lead to increased risks of heart disease (Moss, 2009). Cardiovascular risk factors were investigated in a 2009 study on 470 older adults of all levels of intellectual disability but which had more females and more participants with moderate to severe intellectual disabilities in Holland. Results showed that while over 68% of participants had insufficient exercise levels, central overweight levels of 70.4%, hypertension of 36.8% and diabetes rates of 8.7% were similar to levels seen in the general population. However hypercholesterolemia levels were higher at 31.8% and SB was not investigated (de Winter et al., 2009, 2010). A recent longitudinal study showed that obesity and diabetes were significant predictors of high cholesterol and potentially heart disease in adults with intellectual disability. In this sample 925 participants, 57.2%, completed a 5-year follow-up. Almost 28% of participants were inactive, 36% were obese and 7.1% had diabetes. However, this study did not realise a significant relationship between elevated cholesterol and inactivity but measures of activity were by self-report and doing any form of activity would have registered as being active (1 = inactive, 0 = not inactive) (Murthy and Hsieh, 2021).

A 2012 study investigated the effect of physical inactivity on cardiac atrophy in people with an intellectual disability. Results indicate that cardiac size is significantly smaller in people with an intellectual disability compared to controls and that this cardiac atrophy was acquired through lifestyle habits such as more time in SB. The left ventricular (LV) mass index was inversely correlated with level of intellectual disability and positively related to PA levels and LV stiffness was significantly higher in adults with an intellectual disability compared to the control group. Physical activity levels of people with an intellectual disability were almost 2x less than the controls (*p* < 0.001) (Vis et al., 2012).

### General health

Havercamp et al. (2004) found that compared to adults with no disabilities, adults with intellectual disability were significantly more likely to have fair or poor general health status and be sedentary, with over 33% reportedly having had no exercise in the previous month. However there were no objective measurements or quantifiable amounts of time in activity or SB specified. This 2004 study, which had a large sample size and was representative of all levels of intellectual disability, did not show any significant difference in the obesity or overweight status of the different groups which differs from later studies. Similarly Haider et al. (2013) found that, adults with intellectual disability had SB levels that were almost five times that of the general population (31.1 vs 5.3%), were perceived to have more “fair” or “poor” health, to have diabetes and were significantly more likely to be obese or underweight compared to the general population but concluded that this may be an under-representation due to the intellectual disability participants being identified from an administrative database. A pan-European cross-sectional study assessing the health of people with intellectual disability found that more than half the sample did little or no PA and were classed as sedentary and 41.4% did light activities for about 4 h per week. The study discovered that activity levels decreased with age and the number of people with intellectual disability diagnosed with diabetes, osteoporosis and hypertension increased with age but conclusions were not generalisable as samples were small and unrepresentative of the intellectual disability population in each country (Haveman et al., 2011).

A study assessing the correlates of SB found that physical and mental problems, assessed by a “yes” or “no” answer to a question, were significantly associated with objectively measured SB in a group of 152 adults with intellectual disability of all levels. This average measured sedentary behaviour was found to be 72.9% (Harris et al., 2018).

### Bone mineral density

A study comparing bone mineral density (BMD) levels between individuals with Down syndrome (DS), intellectual disability and non-intellectual disability showed that individuals with DS had significantly less BMD than the other two groups. In addition, both intellectual disability groups had significantly less minutes PA per week but there was only a significant relationship between PA, as determined by walking levels, and BMD levels in the DS group which could pose increased risks of osteoporosis (Geijer et al., 2014).

### Body oxidation

Research has implicated oxidative stress (OS), which is caused by an imbalance between free radical and antioxidant activity, in disease progression (Shruthi et al., 2021). Nitric oxide (NO) is an example of a free radical and is an inflammatory. Although the specificity of serum OS values has been questioned, a study showed significant elevated levels of serum global OS and NO levels in a group of sedentary adults with moderate or profound intellectual disability in residential care, as compared to an active control group (Carmeli et al., 2015). A second study which investigated the differences in plasma total antioxidant status using vitamin and enzyme levels between active and inactive female adults from a residential setting with unspecified intellectual disability levels, found that plasma vitamin levels were significantly lower in the inactive group, indicating that PA could have a protective effect on the antioxidant defence system (Carmeli et al., 2007). Although activity levels were not specified.

## Discussion

Sedentary behaviour (SB) has only recently been recognised as a significant contributor to poor health outcomes (World Health Organisation, 2020). This literature review provides an insight into the emergence of SB as a significant risk factor for multiple diseases that affect adults with an intellectual disability and thus on the overall physical health of this population. It underscores the requirement for detailed studies investigating specific health problems for adults with intellectual disability and their relationship to SB using objective measurements. The results of this literature review have demonstrated that there is a scarcity of research into the effects of SB on the physical health of adults with an intellectual disability, with only eight articles directly investigating the effects of SB on any form of physical health, despite SB prevalence being identified at over 60% in adults with an intellectual disability (Lynch et al., 2021) and the fact that in the general population SB has been shown to have such detrimental effects (López-Valenciano et al., 2020; Owen et al., 2014; Vasankari et al., 2017).

Globally the leading cause of death is ischaemic heart disease and stroke and overall deaths due to diabetes increased by 70% in the 20 years from 2000 to 2019 (Vos et al., 2020; World Health Organisation, 2020). Coincidently levels of SB also increased for European adults in the same timeframe and have been linked to increased disease risk and health problems including cardiovascular disease (CVD), type 2 diabetes, obesity and some cancers (López-Valenciano et al., 2020; Owen et al., 2014; Vasankari et al., 2017). The leading cause of death worldwide is CVD and adults with an intellectual disability have increased risk factors for CVD as demonstrated by significantly higher BMI, greater obesity and inactivity levels, lack of moderate intensity PA, and elevated cholesterol levels (De Winter et al., 2010; Murthy and Hsieh, 2021; Wells et al., 1997; World Health Organisation, 2020). Furthermore, cardiac atrophy, which is a decrease in the myocardial mass, and can be associated with adverse health outcomes, was observed in adults with intellectual disability who exercised significantly less than the controls (Chyrchel et al., 2018; Vis et al., 2012).

According to the US Department of Health, Metabolic syndrome is the term used to describe a cluster of risk factors which can lead to health issues like heart disease and diabetes. Abdominal obesity, high triglyceride levels, high blood pressure and low HDL-cholesterol are examples of risk factors which can contribute to Metabolic syndrome (US Department of Health, 2020). Irrespective of activity level, increased time and fewer breaks in SB were found to be significantly related to a metabolic syndrome rate of 48.6% in a group of US older adults who were sedentary for over 65% of measured time spent wearing an accelerometer (Bankoski et al., 2011). There were no similar studies in the intellectual disability community looking specifically at SB and metabolic syndrome and although higher levels of metabolic syndrome were seen in a Dutch intellectual disability population, no significant relationship was found with PA but SB was not investigated (De Winter et al., 2010). An unsignificant relationship was found between high cholesterol and inactivity despite 36% of the 925 participants being obese while an inverse relationship was observed between levels of diabetes, osteoporosis and hypertension and activity levels as an intellectual disability cohort aged (Haveman et al., 2011; Murthy and Hsieh, 2021). Similarly, the WHO’s definition of metabolic syndrome requires the presence of at least three risk factors i.e. insulin resistance and two risks of either obesity, dyslipidemia or hypertension (Alberti and Zimmet, 1998). While these risk factors are highly prevalent in the intellectual disability population, studies evaluating them collectively are rare so it is difficult to ascertain the actual prevalence of metabolic syndrome. However, obesity and overweight, key risk factors in metabolic syndrome, have been shown to be a critical problem in intellectual disability studies. For example, in Tyrer et al. (2019), 68.5% of intellectual disability participants were either overweight or obese while another intellectual disability study showed 87% of participants were overweight or obese and morbid obesity levels were twice that in the general population (Bryant et al., 2018; Hsieh et al., 2014). Ryan et al. (2021) found that overweight and obesity levels were in excess of 69% in a cohort of older adults with intellectual disability and that those who were obese were nine times more likely to have respiratory issues, which was the largest cause of death for this population (O’Leary et al., 2018). In another study, elevated obesity levels were significantly related to activity levels, but sedentary levels were not investigated (De Winter et al., 2012). Moss (2009) discovered that 67% of their study participants were obese with 28% having elevated glucose levels, while over 85% did not achieve recommended PA levels. Equally, sedentary levels of five times the general population were seen in an intellectual disability cohort who were significantly more likely to be overweight (Haider et al., 2013). A 2004 study by Havercamp and colleagues also found that people with intellectual disability were more sedentary that the controls but conversely there were not significant differences in weight status (Havercamp et al., 2004). Despite the overall negative health effects of obesity, a study found that obesity had a protective effect for osteoporosis, a degenerative disease which increases bone fragility, where obese adults had higher bone mineral density (BMD) than normal-weight participants (Qiao et al., 2020).

Bone mineral density (BMD) is a measure of the strength and integrity of bones and low measures are indicative of the presence of osteoporosis (Kanis et al., 1994; US Department of Health and Human Service, 2004). A cross-sectional study highlighted the poor bone status of a cohort of adults with intellectual disability, where over 30% and 40% had osteopenia and osteoporosis respectively, indicating an increased risk of fracture due to diminished bone integrity (Burke et al., 2019). Another study which compared post-fracture mortality rates between privately insured adults with and without an intellectual disability showed that those with an intellectual disability were at a greater risk of mortality a year after the event occurred (Whitney et al., 2019), emphasising the importance of improving the bone health of adults with an intellectual disability. The National Health and Nutrition Examination Survey (NHANES) study showed a negative association between time spent sedentary and femur BMD, independent of PA level in neurotypical women (Chastin et al., 2014). A study which compared PA and BMD for a group of adults with and without intellectual disability, found that adults with an intellectual disability had lower BMD and that there was a significant relationship between BMD and PA level only in adults with down syndrome (DS), who consequently had an increased risk of osteoporosis (Geijer et al., 2014). While DS is a known contributor to decreased BMD it is a concern that all adults with intellectual disability had low BMD and warrants further investigation of risk factors (Angelopoulou et al., 1999). Thus bone health appears to be an issue for adults with intellectual disability irrespective of aetiology and while a causal relationship has not been explored with SB, it is something that warrants investigation.

Oxidative Stress (OS) is caused by an imbalance in the body whereby an excess of reactive products (free radicals) can lead to cell and tissue damage and consequent progression of diseases like diabetes and hypertension (Pizzino et al., 2017; Taniyama and Griendling, 2003). Two studies investigated the oxidative status of people with intellectual disability, one looking at sedentary behaviour and the other at PA. Comparisons between sedentary and active groups showed raised levels of free radicals were present in the sedentary group (Carmeli et al., 2015). The second study concluded that PA had a protective effect on the body’s antioxidant status when higher plasma vitamin levels were discovered in an active group of adults with intellectual disability compared to an inactive group, although the level of PA was unspecified (Carmeli et al., 2007). Antioxidants such as vitamins and minerals are capable of neutralising these damaging free radicals and protecting the body from disease (Dal and Sigrist, 2016). Unfortunately, the dietary intake for people with intellectual disability has been noted to be poor, with high energy foods and less nutrient-dense selections prevalent, irrespective of their living circumstances, so they may not be getting the required antioxidants from food to combat the negative effects of sedentary behaviour (Adolfsson et al., 2008; Hamzaid et al., 2020).

Multimorbidity is defined as having two or more chronic health issues (van Den Akker et al., 1996). Chronic health conditions, also known as noncommunicable diseases, last for more than 1 year and require ongoing medical care (CDC, 2021). The primary chronic conditions of cardiovascular disease, respiratory disease, cancer and diabetes account for 80% of deaths, with the main contributors of disease coming from the environment, behavioural, physiological and genetic factors (World Health Organisation, 2016). Adults with intellectual disability are at a greater risk of having more chronic health conditions and more likely to lead a sedentary lifestyle (Havercamp et al., 2004). A multimorbidity rate of 71% was observed in the IDS-TILDA longitudinal study, where hypertension and gastrointestinal disease were ubiquitous (McCarron et al., 2013). Sedentary levels were found to be significantly correlated with multimorbidity in a group of adults with intellectual disability while another study showed that over 79% of adults with intellectual disability who had diabetes, also had other health issues like high cholesterol and CVD (Bryant et al., 2018; Tyrer et al., 2019). Although multimorbidity and SB appear to be more widespread in adults with intellectual disability, limited studies have investigated the link, while in the general population these two aspects of health have been studied and a relationship well established (Lewis et al., 2016; Thorp et al., 2011; Vankampfort et al., 2017). In addition, the Irish Longitudinal Study on Aging (TILDA) proposed that a linear dose-dependent association between chronic health conditions and sedentary minutes per day existed (Kandola et al., 2020).

An observation in some studies was that people with a milder intellectual disability who lived more independently were more at risk of unhealthy behaviours and needed more guidance on health risks (de Winter et al., 2009).

People with intellectual disability have significantly poorer health and health outcomes than their peers in the general population (Emerson et al., 2016). In addition, some studies found that people with intellectual disability were more likely to perceive themselves as being in “poor” or “fair” health (Haider et al., 2013; Havercamp et al., 2004). Furthermore, in a group of adults with intellectual disability, perceived physical and mental problems were significantly associated with sedentary levels of 72.9% (Harris et al., 2018). Thus this population’s self-perceived and measured ill-health could be related to elevated sedentary levels. However while the information showing a contributory effect between sedentary behaviour and poorer health in the intellectual disability population is not conclusive, existing studies have shown that higher levels of sedentary behaviour are present in the intellectual disability population, who predominately feel they are not in good health and have more issues being overweight and obese, have excessive chronic health conditions and suffer from metabolic syndrome.

## Conclusion

This literature review has demonstrated that sedentary behaviour (SB) could be a contributor to the poor health which is common in adults with an intellectual disability. The limited studies have shown a prevalence of obesity, multimorbidity and metabolic syndrome as well as elevated levels of SB. However the body of evidence, which is primarily focussed on cross-sectional studies to date does not confirm a cause-and-effect relationship. Further research is required looking specifically at sedentary behaviour and its effect on particular health aspects of adults with an intellectual disability like the excessive obesity levels, multimorbidity and metabolic syndrome. These studies should ideally use objective measurements for accuracy. Thus an in-depth understanding of the health effects of sedentary behaviour on adults with an intellectual disability may be achieved and suitable recommendations made to expediate change, health improvements and consequently improved quality of life for this vulnerable population.
